# Tocilizumab (monoclonal anti-IL-6R antibody) reverses anlotinib resistance in osteosarcoma

**DOI:** 10.3389/fonc.2023.1192472

**Published:** 2023-06-19

**Authors:** Jiuhui Xu, Chenglong Chen, Kunkun Sun, Qianyu Shi, Boyang Wang, Yi Huang, Tingting Ren, Xiaodong Tang

**Affiliations:** ^1^ Musculoskeletal Tumor Center, Peking University People's Hospital, Beijing, China; ^2^ Beijing Key Laboratory of Musculoskeletal Tumor, Peking University People’s Hospital, Beijing, China; ^3^ Department of Orthopedics, Beijing Jishuitan Hospital, Beijing, China; ^4^ Department of Pathology, Peking University People’s Hospital, Beijing, China

**Keywords:** tyrosine kinase inhibitor, interleukin-6, anlotinib, drug resistance, osteosarcoma

## Abstract

**Purpose:**

Anlotinib, a tyrosine kinase inhibitor (TKI) has been in clinical application to inhibit malignant cell growth and lung metastasis in osteosarcoma (OS). However, a variety of drug resistance phenomena have been observed in the treatment. We aim to explore the new target to reverse anlotinib resistance in OS.

**Materials and Methods:**

In this study, we established four OS anlotinib-resistant cell lines, and RNA-sequence was performed to evaluate differentially expressed genes. We verified the results of RNA-sequence by PCR, western blot and ELISA assay. We further explored the effects of tocilizumab (anti- IL-6 receptor), either alone or in combined with anlotinib, on the inhibition of anlotinib-resistant OS cells malignant viability by CCK8, EDU, colony formation, apoptosis, transwell, wound healing, Cytoskeletal stain assays, and xenograft nude mouse model. The expression of IL-6 in 104 osteosarcoma samples was tested by IHC.

**Results:**

We found IL-6 and its downstream pathway STAT3 were activated in anlotinib-resistant osteosarcoma. Tocilizumab impaired the tumor progression of anlotinib-resistant OS cells, and combined treatment with anlotinib augmented these effects by inhibiting STAT3 expressions. IL-6 was highly expressed in patients with OS and correlated with poor prognosis.

**Conclusion:**

Tocilizumab could reverse anlotinib resistance in OS by IL-6/STAT3 pathway and the combination treatment with anlotinib rationalized further studies and clinical treatment of OS.

## 1 Introduction

Osteosarcoma (OS), the most common and severe primary malignant bone tumor, which accounts for approximately 20% of all bone tumors and predominantly arises in the metaphysis of the long bones of children, adolescents, and young adults (1, 2). Despite recent improvements in the therapeutic management of OS, the survival has remained largely unchanged for patients with metastatic or relapsed OS due to its high propensity for pulmonary metastasis at early stage (3–5). The current standard treatment for OS consists of neoadjuvant chemotherapy (pre-operative), extensive surgical resection, and adjuvant chemotherapy (post-operative) (4, 6). OS patients with a surgically respectable tumor may become long-term survivors under standard treatment, while the metastatic, recurrent, and unresectable disease is always fatal (7). Multiple agents that have been testified to be effective in preclinical studies have failed to prolong survival in patients with advanced OS in the past two decades (8–10).

Angiogenesis is a common and pivotal carcinogenic characteristic involved in the progression of OS, especially in advanced OS (5). Numerous reagents are designed to target tumour-associated angiogenesis pathways. Small-molecule anti-angiogenesis tyrosine kinase inhibitors (TKIs) are one of the targeted drugs that specifically inhibit protein tyrosine kinases (PTKs) that are crucial for regulating normal cells within numerous signaling factors and their abnormal activation and dysregulation can cause alterations in tumorigenesis-related downstream signaling (11–13). TKIs have shown more promising prospects compared with other target therapies in OS, with median progression-free and overall survival improved to 4-5 months and 7-11.3 months, respectively (14–16). Anlotinib is a novel orally available multi-targeted TKI that blocks VEGF-R2, PDGFR, EMT, and FGFR and exhibits antitumor activity in several tumors (5, 17). A previous multicenter clinical trial in advanced bone tumors evaluated the safety and anticancer effects of anlotinib, with progression-free survival (PFS) rates of 72.9% and 35.4% at 3 and 6 months respectively, with objective response rates of 7.4% in patients with OS and 37.5% in patients with Ewing’s sarcoma (18). Although anlotinib effectively reduces tumour burden and improves quality of survival and PFS in patients with OS, it is still unchangeable in improving the overall survival of the patients, mainly due to the rapid and inevitable progress of acquired drug resistance (19, 20). For example, in a case of giant delayed pulmonary metastasis of OS treated with anlotinib, the lung metastases in the patient were reduced by 82%, achieving a significant efficacy, but the overall survival of the patient was still only 6.1 months (21). Further immunohistochemical testing of the tumor showed a significant reduction in VEGF-R2 expression after administration of anlotinib, and whole exon sequencing showed mutational amplification of c-MYC, suggesting a possible association with resistance to anlotinib.

In this study, we established acquired anlotinib resistance models in human OS cells and xenograft tumors by exposing gradient increasing concentration of anlotinib. RNA sequencing (RNA-seq) revealed that the interleukin 6 (IL-6) as a prototypical cytokine and cytokine-cytokine receptor interaction pathways are crucial in developing anlotinib resistance in OS cells. IL-6 was first identified in 1973 as a soluble protein produced by T cells that activates the B cell’s differentiation into antibody-producing cells and has been found to be associated with chemotherapy resistance and TKIs resistance in many tumors (22–25). The downstream pathway of STAT3 activated by IL-6 is also believed to be closely related to the proliferation, metastasis, and drug resistance of tumor (10, 26, 27). Here, we explored the effects and mechanisms of tocilizumab, a clinically used drug targeting the IL-6 receptor (IL-6R), combined with anlotinib in treating anlotinib-resistant OS *in vitro* and *in vivo*. Repurposing tocilizumab and other IL-6 inhibitors could be a strategy to treat TKI resistance OS.

## 2 Materials and methods

### 2.1 Cell culture and establishing of anlotinib-resistant cells

The OS cell lines (143B (RRID : CVCL_2270), KHOS (RRID : CVCL_2546), MG-63 (RRID : CVCL_0426), U2OS (RRID : CVCL_0042)) were purchased from the American Type Culture Collection (ATCC). All the cell lines have been authenticated within the last three years and were mycoplasma-free. 143B cells were cultured in DMEM (Gibco), KHOS, MG63, U2OS cells were cultured in RPMI-1640 (Gibco), with 10% fetal bovine serum (FBS, Wisent) and 1% penicillin/streptomycin (PS, Gibco). All cells were cultured in the incubator at 37 °C in humidified air with 5% CO^2^ and stored in CELLSAVING solution (New Cell & Molecular Biotech) at −80°C. Anlotinib (S8726) and Tocilizumab (anti-human IL-6R, A2012) were both purchased from Selleckchem. All products were stored and used according to manufacturer’s instructions.

To establish anlotinib-resistant cells, we treated the KHOS, 143B, MG63 and U2OS cells with gradually increasing anlotinib concentrations from 0.5 to 5 μM over 5 months. The culture medium was changed and anlotinib was added daily. Once the cells maintained stable proliferationin the existing anlotinib concentration, the anlotinib concentration was increased in with an 0.5 μM increments. Half maximal inhibitory concentrations (IC_50_) were tested by CCK8 assay to detect cellular sensitivity to anlotinib-resistant cells (KHOS-R, 143B-R).

### 2.2 CCK8 assay

The OS cells and anlotinib-resistant cells were seeded into 96-well plates at a density of 3× 10^3^ cells/well. The cells were treated with different anlotinib concentrations and 100ng/ml tocilizumab. After 24-hour treatment, Cell Counting Kit-8 (CCK8, Dojindo) were added into culture medium following the manufacturer’s instructions. The cells were incubated for 1 hour in incubator, and the optical density (OD) value was measured at 450 nm using a microplate reader (Bio- Rad). IC_50_ of OS cells to anlotinib were calculated using GraphPad Prism 9.0.

### 2.3 Colony formation assay

The cells were seeded into six-well plates at a concentration of 800 cells/well, incubated overnight, subsequently treated with 3μM anlotinib and 100ng/ml tocilizumab. The culture medium was changed every three days. After one week of treatment, the cells were fixed with 4% fixative solution (P1110, Solarbio) and stained with 0.1% crystal violet stain solution (G1063, Solarbio).

### 2.4 Apoptosis assay

The OS cells were seeded into six-well plates at a concentration of 5×10^5^ cells/well, incubated overnight, and treated with different drugs at the mentioned concentrations for 24 h. Then the cells were stained with the FITC Annexin V Apoptosis Detection Kit I (556547, BD Biosciences) following the manufacturer’s instructions, and examined by flow cytometer (C6 Plus, BD Biosciences).

### 2.5 RNA sequencing

RNA was isolated from 4 kinds of OS cells and corresponding resistant OS cells, RNA sequencing (RNA-Seq) was performed by Biomarker Technologies Corporation (Beijing, China). The cDNA library was constructed using the NEBNext Ultra RNA Library Prep Kit for Illumina (NEB, E7530) and NEBNext Multiplex Oligos for Illumina (NEB, E7500). Gene expression levels were estimated using fragments per kilobase of exon per million fragments mapped (FPKM) values using the Cufflinks software. Differentially expressed genes (sensitive vs. resistant) were identified as log2 (fold change) > 2 and adjusted *P* value <0.05.

### 2.6 RNA isolation and qRT-PCR assay

Total RNA was isolated using TRIzol reagent (15596018, Invitrogen) following the manufacturer’s protocol. Complementary DNA (cDNA) was synthesized using PrimeScript RT Master Mix (TaKaRa Biotechnology). qRT-PCR was performed to measure the relative target gene expressions using SYBR Green premix Ex Taq (TaKaRa, RR420A). Data were normalized to the controls and measured using the comparative CT method. The sequence primers were listed in **Supplementary Table 1**.

### 2.7 Enzyme-linked immunosorbent assay

The cell culture supernatants of OS cells and anlotinib-resistant OS cells were collected to be used immediately or stored at -80°C. ELISA was performed by following the manufacturer’s instructions using enzyme‐linked immunosorbent assay kit (70-EK106, Multiscience).

### 2.8 Protein extraction and western blot

Total protein was extracted from cell lysates using cell lysis buffer (Cell Signaling Technology). The protein concentration was measured using the BCA Protein Assay kit (Solarbio). Then, protein was loaded into SDS- PAGE gels and transferred onto PVDF membranes, and blocked with rapid Closure Solution (WB1601, Biotides) for 20min. The membranes were incubated with primary antibodies including anti-IL6 (77888-1-Ig, Proteintech), anti-N-cadherin (22018-1-AP, Proteintech), anti-Vimentin (60330-1-Ig, Proteintech), anti-STAT3 (10253-2-AP, Proteintech), anti-GAPDH (60004-1-Ig, Proteintech) and anti-β-actin (ab8226, Abcam) at 4 °C overnight. The next day, the secondary antibodies were added onto the membranes at 37 °C for 1 h. Protein bands were visualized using the Image Lab Software (Bio-Rad).

### 2.9 5-ethynyl-2’ -deoxyuridine assay

KHOS-R cells was added into 12 well plates and incubated overnight. Then, the cells were treated with 3μM anlotinib or 100ng/ml tocilizumab for 24h. EdU assay was performed using BeyoClick™ EdU Cell Proliferation Kit with Alexa Fluor 488 (C0071S, Beyotime Biotechnology) following the manufacturers’ protocol. Stained cells were photographed using a fluorescence microscope (Leica).

### 2.10 Wound healing assay

The cells were plated into a six-well plate and incubated until reached 80% confluence. A sterile pipette tip was used to scratch a line on the cells. Cells were washed twice with PBS and then treated with 3μM anlotinib or 100ng/ml tocilizumab for 24h.The images were taken at 0 hour and 24 hours under the microscope, the cell migration area was measured using Image J software.

### 2.11 Transwell assay

Transwell inserts (3422, Corning, NY, USA) were used for our transwell assay. Cell complete medium with 3μM anlotinib was added into the bottom chamber of transwell plate, 1.2×10^4^ cells were seeded into the upper chamber and incubated with 100ng/ml tocilizumab for 24 hours. The migrating cells on the underside of the inserts were fixed with 4% fixative solution and stained with 0.1% crystal violet for 15 min. The cells remained on the upper chamber membrane were removed gently with a cotton swab. Images were acquired by the microscope, and the number of migrating cells was measured using Image J software.

### 2.12 Cytoskeletal stain assay

The anlotinib-resistant cells were added into 24 well plates and incubated overnight. Then, the cells were treated with 3μM anlotinib or 100ng/ml tocilizumab for 24h, and fixed with 4% fixative solution. TRITC Phalloidin (CA1610, Solarbio) was used for cytoskeletal stain assay and cell nuclei were stained with DAPI (Solarbio) following the manufacturers’ protocol. Images were photographed using a fluorescence microscope.

### 2.13 Immunohistochemistry

We collected 104 osteosarcoma tissues after surgery, which were approved by the Ethics Committee of the Peking University People’s Hospital (2021PHE030), and made into 3 tissue arrays. Tumor tissues excised from the mice were fixed in 4% paraformaldehyde immediately, embedded in paraffin, and cut into 4-μm sections. The methods of Immunohistochemistry (IHC) and immunoreactive score (IRS) evaluation were performed as previously described (5). The Paraffin sections were reacted with the anti-IL-6 antibody.

### 2.14 Orthotopic osteosarcoma mouse model construction

The orthotopic osteosarcoma mouse models were performed using BALB/c-nude mice (female, 6-week-old). Animal experiments were approved by the ethics committee of Peking University People’s Hospital. To establishing the xenograft tumor model, 10μL 10^6^ anlotinib-resistant 143B-R cells were implanted into mice fibular marrow cavity and then randomly divided into four groups (4 mice per group): NC, anlotinib, tocilizumab, and combination therapy groups. Anlotinib was gastric gavage administration at a concentration of 2mg/kg once per day for 14 consecutive days, tocilizumab was injected at a concentration of 5mg/kg every one week intraperitoneally. The mice were treated once the obvious tumor formation, after 14-day treatment, the mice were sacrificed, the lungs and legs with tumor were removed, tumors and lungs were stored in 4% paraformaldehyde for IHC and HE staining analysis.

### 2.15 Statistical analysis

Data was expressed as mean ± S.D. (standard deviation). Unpaired two-tailed Student t test was used for analysis of two samples. The One- way ANOVA was used among different groups. All statistical analysis were performed with the GraphPad Prism 9.0. The level of significance was chosen as P < 0.05.

## 3 Results

### 3.1 Construction and validation of anlotinib-resistant osteosarcoma cells

**Figure 1:**
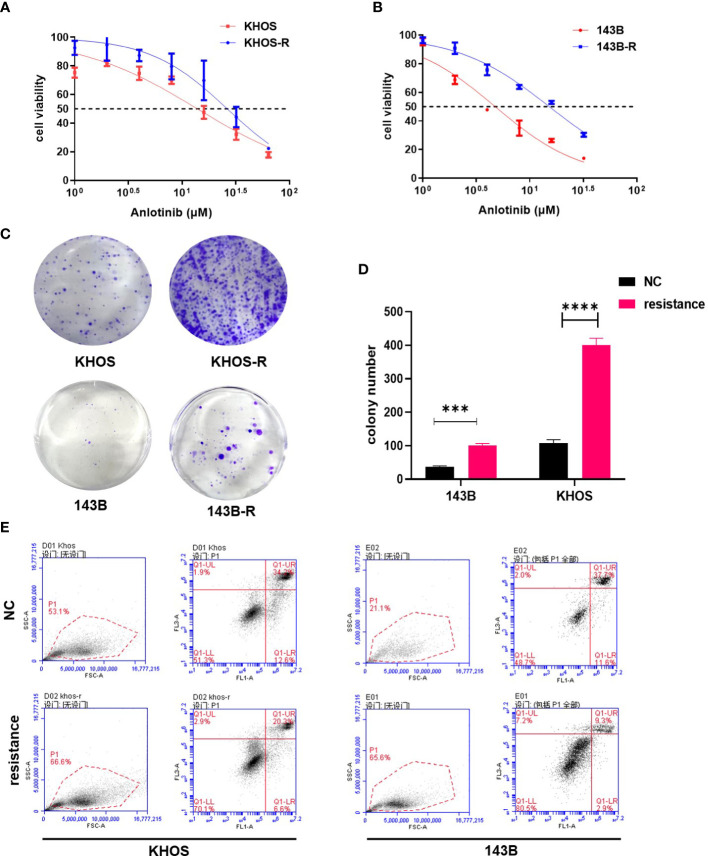
The validation of established anlotinib-resistant OS cells. **(A)** The results of CCK8 assay showed IC_50_ values of KHOS cells and KHOS-R were13.91μM and 26.38μM respectively. **(B)** The IC_50_ value of 143B cells to anlotinib is 4.758μM, and 143B-R cells is 15.25 μM. **(C)** The colony formation results indicated KHOS-R and 143B-R were more insensitive to anlotinib. **(D)** The quantified data of colony formation assay. **(E)** The results of apoptosis showed both KHOS-R and 143B-R cells were more insensitive to anlotinib for 24h treatment. (***P < 0.001, ****P < 0.0001).

In order to construct anlotinib-resistant osteosarcoma cell lines, we gradually increased the anlotinib concentration added in the culture medium of 143B, KHOS, MG63 and U2OS cells over 4 months. To verify the establishment of the anlotinib-resistant cells, we first compared the IC_50s_ of OS cells with the anlotinib-resistant OS cells for 24 h by CCK8 assay. We found that the IC_50s_ of 143B and KHOS were 4.758μM and 13.91μM respectively, while the IC_50s_ of 143B-R and KHOS-R were increased to 15.25 μM and 26.38μM respectively (**Figures 1A, B**). In addition, the clone formation results showed that the proliferation of OS cells were obviously inhibited in response to 3 μM anlotinib, while the 143B-R and KHOS-R cells were not sensitive to anlotinib (**Figures 1C, D**). The results of apoptosis also showed that anlotinib-resistant OS cells significantly down-regulated their sensitivity to anlotinib (**Figure 1E**). All these results indicated the anlotinib-resistant OS cell lines were successfully constructed.

### 3.2 IL-6/STAT3 axis was the key pathway in OS anlotinib resistance

**Figure 2:**
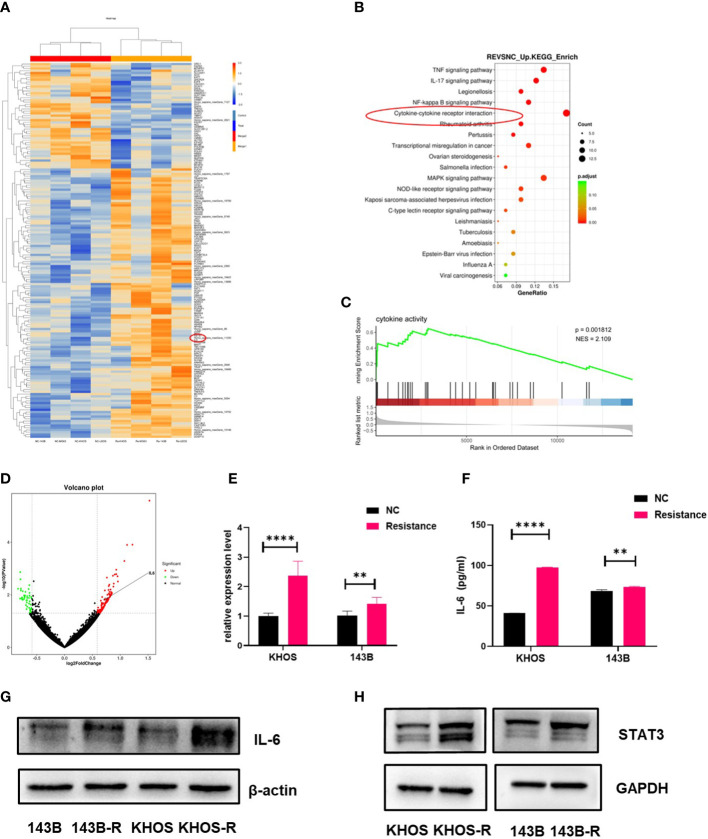
IL-6/STAT3 axis played a critical role in OS anlotinib resistance. **(A)** The heatmap showed the differentially-expressed genes between NC (OS cell lines) and RE (anlotinib-resistant cell lines). IL-6 was up-regulated in anlotinib-resistant cells (143B-R, KHOS-R, MG63-R, U2OS-R). **(B)** KEGG enrichment analysis showed the differentially-expressed enrichment signaling pathways between OS cell lines and anlotinib-resistant OS cells. Cytokine-cytokine receptor interaction was highly active in anlotinib-resistant OS cells (143B-R, KHOS-R, MG63-R, U2OS-R). **(C)** Gene set enrichment analysis (GSEA) validated enrichment of cytokine activity in anlotinib-resistant OS cell lines. **(D)** The volcano plot showed IL-6 was up-regulated in anlotinib-resistant OS cell lines. **(E)** The qPCR results showed IL-6 mRNA expression was both increased in KHOS-R and 143B-R cells when compared with OS cell lines. **(F)** The ELISA assay results showed IL-6 secretion was both increased in KHOS-R and 143B-R cells when compared with OS cell lines. **(G)** Western blot results indicated IL-6 protein expression was up-regulated in 143B-R and KHOS-R cells. **(H)** The results of Western blot showed STAT3 protein was increased in anlotinib resistant cells which revealed STAT3 pathway was activated. (**P <0.01, ****P < 0.0001).

OS cell lines (negative control, NC) and anlotinib-resistant cell lines (RE) were collected for second-generation RNA sequencing, and the differentially-expressed genes between NC and RE were shown in **Figure 2A**, we found cytokine-cytokine receptor interaction was highly activated by KEGG enrichment analysis (**Figure 2B**). Gene set enrichment analysis (GSEA) further validated the enrichment of cytokine activity in anlotinib-resistant OS cell lines (**Figure 2C**). We found that IL-6 expression was significantly increased in anlotinib-resistant OS cells (**Figures 2A, D**), which suggested that IL-6 might be a key cytokine promoting anlotinib resistance. qPCR, western blot and ELISA assays were performed to further validate the sequencing results. Unexpectedly, the results further showed IL-6 was all enhanced in mRNA, intracellular protein and secretory protein levels (**Figures 2E–G**). IL-6 acts directly at IL-6R located on tumor cells to induce the expression of STAT3 target genes, and then drive tumor proliferation, survival and drug resistance (28). According to the results of western blot, we found the STAT3 expression was up-regulated in anlotinib-resistant cells (**Figure 2H**). These results revealed that IL-6/STAT3 axis was the key pathway in OS anlotinib resistance.

### 3.3 Combination with tocilizumab attenuated the survival of osteosarcoma-resistant cells

**Figure 3:**
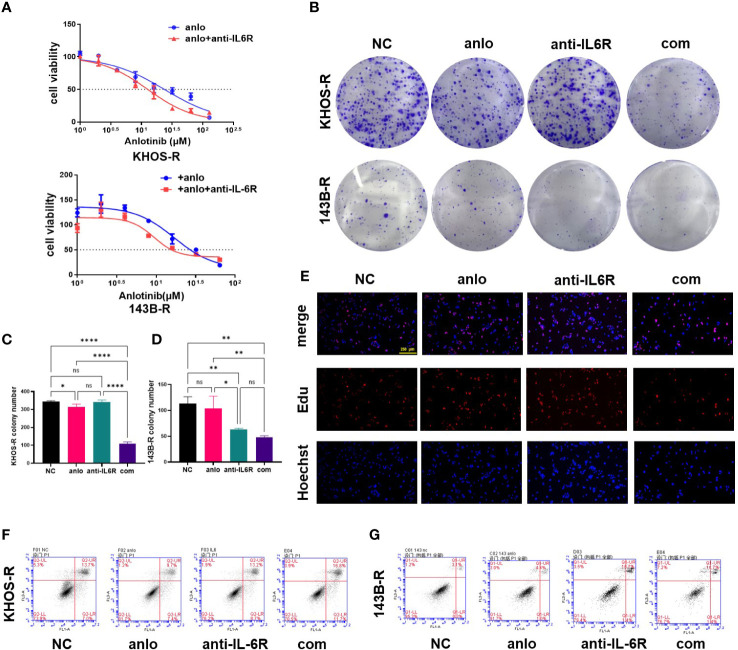
Combination with tocilizumab inhibited the proliferation of anlotinib-resistant cells. **(A)** The IC_50_ values of combination treatment were decreased to 12.70μM and 9.388μM from 23.76μM and 16.91μM for KHOS-R and 143B-R cells respectively. **(B)** The number of colony formation was significantly inhibited in combination treatment group of anlotinib resistant cells, and tocilizumab monotherapy showed anti-tumor function on 143B-R cells. **(C, D)** The statistical analysis of colony formation results of KHOS-R and 143B-R cells. **(E)** Representative pictures from Edu assay. The results showed combination treatment obviously inhibited the proliferation viability of KHOS-R cells. **(F, G)** The results of apoptosis assay showed combination treatment obviously promoted anlotinib-resistant OS cells apoptosis. (ns, no significance; *P<0.05, **P<0.01, ****P<0.0001).

The 24-hour IC_50s_ of resistant cells were first tested by CCK8 assay. The results showed the IC_50_ values of anlotinib monotherapy for KHOS-R and 143B-R were 23.76μM and 16.91μM, combined with 100ng/ml tocilizumab (an IL-6R inhibitor) (29), the IC_50s_ were reverted to 12.70μM and 9.388μM respectively (**Figure 3A**). The cell proliferation viability results indicated that 100ng/ml tocilizumab affected proliferation of resistant cells lightly, but combination with anlotinib obviously diminished proliferation viability and regained sensitivity to anlotinib according to colony formation and EDU assays (**Figures 3B–E**). The effect of combination treatment on OS cells apoptosis was also tested, in KHOS-R cells, we found tocilizumab monotherapy was ineffective, while a significant apoptosis rate was showed in combination therapy (**Figure 3F**). As for 143B-R cells, no obvious apoptosis difference was shown between the NC and anlotinib groups, and low dose tocilizumab or combination therapy both upregulated apoptosis rates of 143B-R (**Figure 3G**).

### 3.4 Combination therapy was more effective to suppress the malignant progression of resistant cells by blocking the IL-6/STAT3 axis

**Figure 4:**
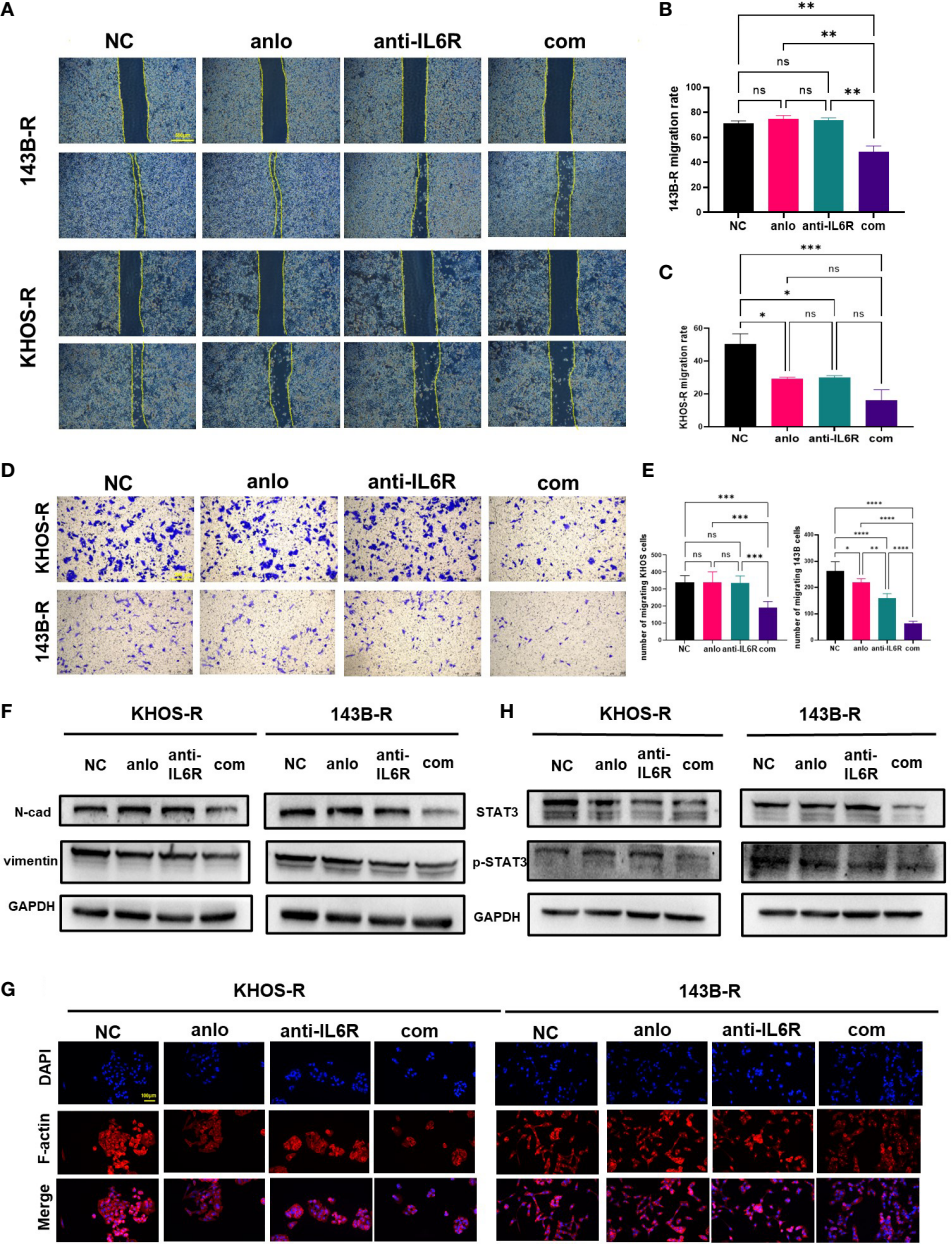
Combination therapy was more effective to suppress OS malignant progression by blocking IL-6/STAT3 axis. **(A)** The migration ability of anlotinib-resistant OS cells was significantly inhibited by the combination treatment with tocilizumab. **(B, C)** The statistical plots showed the combination treatment with tocilizumab decreased the healing rate of anlotinib-resistant cells, and migration ability of anlotinib-resistant KHOS cells could also be inhibited by tocilizumab monotherapy. **(D)** The results of transwell assay showed the migration ability of anlotinib-resistant OS cells was significantly inhibited by the combination treatment. **(E)** The statistical data of transwell assay for 143B-R and KHOS-R cells. **(F)** Western blot results showed the EMT-related protein N-cad expression and vimentin expression were decreased which was caused by combination treatment. **(G)** The combination treatment with tocilizumab inhibited cytoskeleton formation of anlotinib-resistant OS cells. **(H)** Western blot results showed the STAT3 expression and phosphorylation were inhibited because of the combination treatment. (ns, no significance; *P < 0.05, **P < 0.01, ***P < 0.001, ****P < 0.0001).

Malignant cancer progression also encompasses the tumor migration and invasion, which closely related with tumor metastasis (30). The results of wound healing and transwell assays showed combination therapy was more effective to diminished tumor migrating and invasive ability (**Figures 4A–E**). Epithelial-mesenchymal transition (EMT) has been defined as a process which epithelial cells lose the epithelial features and acquire mesenchymal features. For a long time, EMT has been viewed associating with various tumor functions, including tumor cell migration, metastasis, and resistance to therapy (31). Combination with tocilizumab treatment significantly downregulated mesenchymal marker proteins including N-cadherin and Vimentin, which indicated combination with tocilizumab inhibited EMT of OS resistant cells (**Figure 4F**). Cytoskeletal structure is useful to maintain cell physiological activity and tissue homeostasis (32). F-actin staining was used to assess the effect of tocilizumab on the anlotinib resistant cell, and the results showed anlotinib combinated with tocilizumab obviously impaired the cytoskeletal structure (**Figure 4G**). We have found the STAT3 expression was upregulated in anlotinib-resistant cells as mentioned, whether IL-6/STAT3 pathway can be inhibited by tocilizumab was not clear. The results of western blot showed combination therapy decreased STAT3 expression in 143B-R cells. Meanwhile, 100ng/ml tocilizumab monotherapy and combination therapy were both able to inhibit IL-6/STAT3 pathway in KHOS-R cells (**Figure 4H**).

### 3.5 Tocilizumab enhanced the antitumor effect of anlotinib *in vivo*

**Figure 5:**
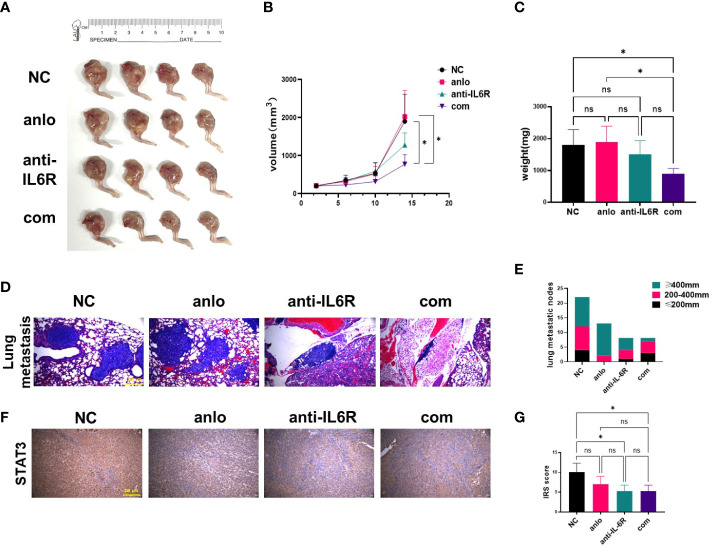
Tocilizumab enhanced the antitumor effect of anlotinib *in vivo*. **(A)** Xenograft photographs were taken after 14-day administration with anlotinib monotherapy, tocilizumab monotherapy and combination therapy. **(B)** Growth curves of tumors presented by volume evaluated once 4 days. **(C)** The results of tumor weights at the day of mice were euthanized. **(D)** Representative images of lungs harvested from mice after 14 days of treatment. **(E)** The statistical analysis results of pulmonary metastasis. **(F)** Representative STAT3 staining of tumor sections from mouse models were shown. **(G)** Quantification of STAT3 expression was performed in different treatment groups. (ns, no significance; *P < 0.05).

Orthotopic xenograft model of OS in nude mice was established employing the 143B-R cells. The obvious tumor formation was shown, and then treatments of different groups were performed. After 14-day treatment, we found combination therapy with tocilizumab significantly inhibited tumor growth, as shown by the results of the volume and weight of the tumor tissues (**Figures 5A–C**). The images of the lungs showed that tocilizumab monotherapy and combination therapy both decreased the number of pulmonary metastatic nodules. And combination therapy significantly inhibited the growth of osteosarcoma lung metastasis nodules compared with NC group and anlotinib treatment group by HE staining (**Figures 5D, E**). To detect whether STAT3 pathway was blocked in mouse models, STAT3 expression levels in different treatment groups were examined by IHC assay. And we found STAT3 expressions were all decreased in anlotinib group, anti-IL-6R group, and combination treatment group (**Figures 5F, G**).

### 3.6 IL-6 was highly expressed in patients with osteosarcoma and correlated with poor prognosis

**Figure 6:**
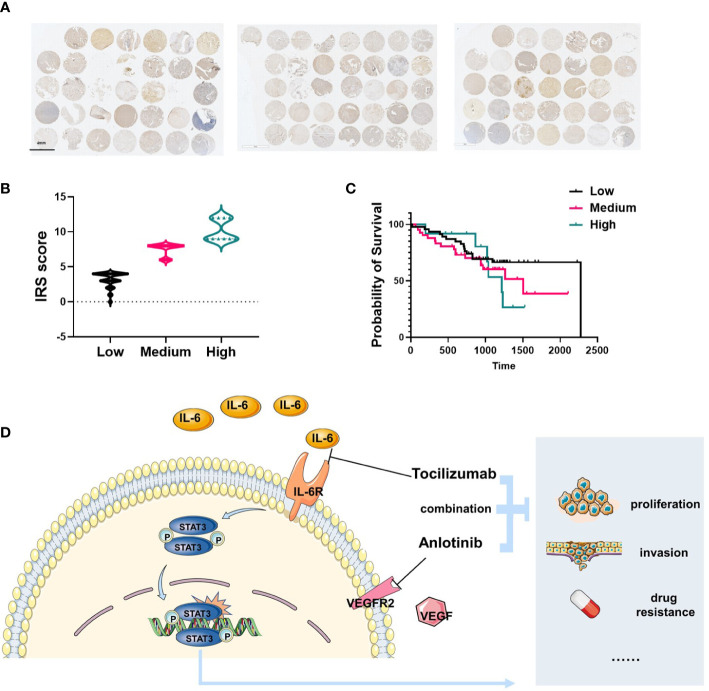
IL-6 was highly expressed in patients with osteosarcoma and correlated with poor prognosis. **(A)** Total 104 tissues from OS patients were collected and IL-6 expression was shown by IHC. **(B)** The IRS score of IL-6 expression was determined and translated into three levels: high (IRS 9–12), medium (IRS 5–8), and low (0–4). **(C)** The survival analysis indicated the patients with high level IL-6 expression were correlated with poor prognosis (n=104). **(D)** Schematic diagram illustrating the hypothetic mechanisms of combination therapy with tocilizumab enhances sensitivity to anlotinib for anlotinib resistant OS cells.

Tissue microarrays were established using 104 tumor specimens, and were used to examine IL-6 expression in patients with osteosarcoma by IHC staining (**Figure 6A**). We found IL-6 was highly expressed in OS tissues, and we translated IRS scores into three levels: high, medium, and low groups (**Figure 6B**). Combined with patient clinical information, we found that IL-6 expression was correlated with poor prognosis of patients, the median survival of the patients in high, medium, and low IL-6 expression groups were 1217, 1508, and 2274 days respectively (**Figure 6C**). **Figure 6D** presented the hypothetic mechanisms of combination therapy with tocilizumab enhances sensitivity to anlotinib for anlotinib resistant OS cells.

## 4 Discussion

Treatment for patients with advanced OS and other malignancies has been unsatisfactory, even a short-term response to TKIs is initially achieved (5, 33–35). Nearly all patients succumb to relapse because of drug resistance, for which no effective therapy is available at present. In addition to the rapid and inevitable development of drug resistance, the side effects of TKIs during treatment are also important factors that prevent patients from maintaining effective treatment (20). Here we first demonstrated that the IL-6/STAT3 pathway is activated in human OS anlotinib-resistant cells. We first established anlotinib-resistant subtype OS cells by exposing the cells to gradually increasing doses of anlotinib for over 5 months as we previously published the study, and the cells were grown *in situ* in nude mice for a stable orthotopic OS mouse model (5). Through this model combined with the results of second-generation sequencing, increased expression of IL-6 and robust activation of the cytokine-cytokine receptor interaction signaling pathway were identified in the anlotinib-resistance subset when compared with their parental sensitive cells, indicating that the IL-6 plays a crucial role in the progression of developing resistance to anlotinib. It has been reported that the increased secretion of IL-6 can induce resistance in non-small cell lung cancer cells to osimertinib, a third-generation EGFR-TKI (36). In a similar study, Li et al. demonstrated that inhibition of IL-6 signaling can sensitizes TKI-resistant human lung cancer cells, suggesting that IL-6 might be a breakthrough in addressing TKI resistance (37). In this study, we verified the high expression of IL-6 in resistance OS cells relative to sensitive cells at transcriptome and protein levels, respectively, using q-PCR, western blot, and ELISA assays. This is consistent with other studies that have reported high expression of IL-6 in OS (26, 38, 39). In addition, we further verified the biological variances between resistant cells and sensitive cells by using colony formation assay and apoptosis assay to confirm the successful establishment of anlotinib-resistant OS cells.

In our cell viability assay, the IC_50_ values of anlotinib monotherapy for KHOS-R and 143B-R cells were shown to be 23.76 μM and 16.91 μM respectively, which were 1.71 folds and 3.56 folds higher than those of their parental sensitive cells. However, with tocilizumab treatment, the IC_50_ values of resistance cells were reverted to 12.70 μM and 9.38 μM respectively, suggesting that tocilizumab administration could reverse the resistance and be of therapeutic value in OS. We verified our hypothesis by evaluating the proliferation, invasion, migration, apoptosis, and cytoskeleton formation of resistance cells after administration with anlotinib, tocilizumab, and a combination treatment. The results suggested that tocilizumab effectively overcomes TKI resistance in established TKI-resistant cell lines. These cellular biological functions are unlikely to be solely related to IL-6 expression but are usually directly induced and regulated by its downstream proteins and pathways. It is well known that aberrant overexpression of IL-6 drives the formation of the tumor microenvironment, and OS tumor cells produce large amounts of soluble IL-6, accompanied by increased phosphorylation of STAT3, and exhibit strong chemotaxis to those factors (10, 40). STAT3 is constitutively triggered during the onset and development of OS by tyrosine phosphorylation by a range of signaling pathways, including cytokines from the IL-6 family, and its overexpression is associated with resistance to chemotherapeutics and decreased survival of the patients, and emerges as a potential therapeutic target (10, 26, 41). In this study, high level of STAT3 in resistance cells (143B-R, KHOS-R, MG63-R, and U2OS-R cell lines) compared with their parental cells was verified through western blot. This suggests the biological properties of resistant OS cells such as improved proliferation, invasion, and migration abilities, may be achieved through IL-6-induced up-regulation of STAT3.

When combined anlotinib with tocilizumab for blocking the IL-6R expression, the neoplastic features of OS cells were significantly suppressed compared with anlotinib monotherapy. In addition, we tested the protein expression of STAT3 in different drug administration groups of cells, and the results were consistent with our hypothesis that neither anlotinib monotherapy nor IL-6R monotherapy reduced STAT3 expression in resistant cells, while the combination treatment strikingly restrained the STAT3 expression and phosphorylation. Besides the inhibitory effect on the IL-6/STAT3 signaling pathway, tocilizumab’s ability to reverse EMT may also play an important role in overcoming anlotinib resistance. In our further study, similar trends are observed in N-cad expression, an epithelial-mesenchymal transition-related protein, and in vimentin expression that is strongly connected with cytoskeleton formation and metastasis in tumors. This also confirms the therapeutic effect of combining tocilizumab and anlotinib on the invasion, metastasis and cytoskeleton formation of OS-resistant cell at the molecular level.

Powerful therapeutic effects of tocilizumab were also observed in our anlotinib-resistant xenograft nude mouse model. These results are consistent with those of previous studies investigating the role of the IL-6/STAT3 pathway in lung cancer (37, 42). Meanwhile, inhibiting IL-6/STAT3 suppressed tumor cell growth and enhanced the sensitivity to antitumor drugs (37, 43). For further exploring the correlation between IL-6 expression and the prognosis of patients with OS, we performed microarray analysis and survival analysis in 104 patients with OS, and our results were consistent with previous studies that patients suffering from OS with a poor prognosis are likely to have high IL-6 expressed (44, 45). Therefore, IL-6 expression can be used as an important prognosis predictor in OS patients.

However, there are some limitations still existed. IL-6/STAT3 signaling pathway contains many other molecules, and we just validated the STAT3 expression and its phosphorylation, other molecules expression may be further explored. Our results showed IL-6/STAT3 signaling pathway was the correlated with anlotinib resistance and patients’ poor prognosis. However the clinical information about the anlotinib efficacy in patients with OS was not gotten and further analyzed.

In conclusion, we found that IL-6 expression was upregulated in anlotinib resistant OS cell lines and tocilizumab could decrease IL-6/STAT3 signaling activation and reverse EMT. Notably, administering tocilizumab or IL-6 inhibitor combined with anlotinib, as a new therapeutic option, can overcome anlotinib-resistant tumor progression. Meanwhile, the side effects of anlotinib and other TKIs such as oral mucositis, low platelet count, low white blood cell count, and abnormal liver function may be mitigated by combining with tocilizumab (20). Most combinational therapeutic strategies achieved better efficacy than single-drug administration in tumors. However, finding combination regimens based on resistance mechanisms is tough but crucial for heterogeneous tumors such as OS. The present study provided a potential combination strategy to treat OS by examining the putative mechanisms of anlotinib resistance and demonstrating an effective way to overcome this resistance. Further studies should be conducted to validate the clinical efficacy of tocilizumab or other IL-6 inhibitors for anlotinib resistant OS management.

## Data availability statement

The raw data supporting the conclusions of this article will be made available by the authors, without undue reservation.

## Ethics statement

The studies involving human participants were reviewed and approved by Ethics Committee of the Peking University People’s Hospital. The patients/participants provided their written informed consent to participate in this study. The animal study was reviewed and approved by ethics committee of Peking University People’s Hospital.

## Author contributions

JX: Investigation, Methodology, Writing, Original Draft; CC: Conceptualization, Methodology, Writing; KS: Validation, Formal analysis; QS: Data curation, Software; BW: Data curation, Editing;YH:Editing; TR: Supervision, Validation; XT: Resources, Supervision, Funding acquisition. All authors contributed to the article and approved the submitted version.
