# Supplementary material for: Tocilizumab (monoclonal anti-IL-6R antibody) reverses anlotinib resistance in osteosarcoma
Source: Front Oncol. 2023 Jun 19;13:1192472. doi: 10.3389/fonc.2023.1192472 (PMC10315670; doi:10.3389/fonc.2023.1192472)
Supplement: Supplementary file 1 [file Table_1.docx]

**SUPPLEMENTAL INFORMATION**

**Tocilizumab (monoclonal anti-IL-6R antibody) reverses anlotinib resistance in osteosarcoma**

| Table S1 The primer of qPCR | | |
| --- | --- | --- |
| **Gene** | **Forward** | **Reverse** |
| IL-6 | AGACAGCCACTCACCTCTTCAG | TTCTGCCAGTGCCTCTTTGCTG |
| GAPDH | GTCTCCTCTGACTTCAACAGCG | GTCTCCTCTGACTTCAACAGCG |
| IL-1β | CCACAGACCTTCCAGGAGAATG | GTGCAGTTCAGTGATCGTACAGG |

Supplementary Table 1：
